# The Neutrally Charged Diarylurea Compound PQ401 Kills Antibiotic-Resistant and Antibiotic-Tolerant Staphylococcus aureus

**DOI:** 10.1128/mBio.01140-20

**Published:** 2020-06-30

**Authors:** Wooseong Kim, Guijin Zou, Wen Pan, Nico Fricke, Hammad A. Faizi, Soo Min Kim, Rajamohammed Khader, Silei Li, Kiho Lee, Iliana Escorba, Petia M. Vlahovska, Huajian Gao, Frederick M. Ausubel, Eleftherios Mylonakis

**Affiliations:** a College of Pharmacy, Graduate School of Pharmaceutical Sciences, Ewha Womans University, Seoul, Republic of Korea; b Institute of High Performance Computing, A*STAR, Singapore, Singapore; c Division of Infectious Diseases, Rhode Island Hospital, Warren Alpert Medical School of Brown University, Providence, Rhode Island, USA; d Department of Molecular Physiology and Biological Physics, University of Virginia School of Medicine, Charlottesville, Virginia, USA; e Department of Mechanical Engineering, Northwestern University, Evanston, Illinois, USA; f Department of Engineering Sciences and Applied Mathematics, Northwestern University, Evanston, Illinois, USA; g School of Mechanical and Aerospace Engineering, College of Engineering, Nanyang Technological University, Singapore, Singapore; h Department of Molecular Biology, Massachusetts General Hospital, Boston, Massachusetts, USA; i Department of Genetics, Harvard Medical School, Boston, Massachusetts, USA; Rutgers University

**Keywords:** persisters, antibiotic tolerance, antimicrobial resistance, MRSA, membrane-active antimicrobials, antibiotic, bacterial persister, *Caenorhabditis elegans*, membrane-active agent

## Abstract

Resistance or tolerance to traditional antibiotics is a challenging issue in antimicrobial chemotherapy. Moreover, traditional bactericidal antibiotics kill only actively growing bacterial cells, whereas nongrowing metabolically inactive cells are tolerant to and therefore “persist” in the presence of legacy antibiotics. Here, we report that the diarylurea derivative PQ401, previously characterized as an inhibitor of the insulin-like growth factor I receptor, kills both antibiotic-resistant and nongrowing antibiotic-tolerant methicillin-resistant Staphylococcus aureus (MRSA) by lipid bilayer disruption. PQ401 showed several beneficial properties as an antimicrobial lead compound, including rapid killing kinetics, low probability for resistance development, high selectivity to bacterial membranes compared to mammalian membranes, and synergism with gentamicin. In contrast to well-studied membrane-disrupting cationic antimicrobial low-molecular-weight compounds and peptides, molecular dynamic simulations supported by efficacy data demonstrate that the neutral form of PQ401 penetrates and subsequently embeds into bacterial lipid bilayers more effectively than the cationic form. Lastly, PQ401 showed efficacy in both the Caenorhabditis elegans and Galleria mellonella models of MRSA infection. These data suggest that PQ401 may be a lead candidate for repurposing as a membrane-active antimicrobial and has potential for further development as a human antibacterial therapeutic for difficult-to-treat infections caused by both drug-resistant and -tolerant S. aureus.

## Abstract (precis)

Membrane-damaging antimicrobial agents have great potential to treat multidrug-resistant or multidrug-tolerant bacteria against which conventional antibiotics are not effective. However, their therapeutic applications are often hampered due to their low selectivity to bacterial over mammalian membranes or their potential for cross-resistance to a broad spectrum of cationic membrane-active antimicrobial agents. We discovered that the diarylurea derivative compound PQ401 has antimicrobial potency against multidrug-resistant and multidrug-tolerant Staphylococcus aureus. PQ401 selectively disrupts bacterial membrane lipid bilayers in comparison to mammalian membranes. Unlike cationic membrane-active antimicrobials, the neutral form of PQ401 rather than its cationic form exhibits maximum membrane activity. Overall, our results demonstrate that PQ401 could be a promising lead compound that overcomes the current limitations of membrane selectivity and cross-resistance. Also, this work provides deeper insight into the design and development of new noncharged membrane-targeting therapeutics to combat hard-to-cure bacterial infections.

## INTRODUCTION

Staphylococcus aureus is a Gram-positive bacterial pathogen that colonizes the skin or nasal cavity of approximately one-third of the human population (1). S. aureus is one of the most significant human bacterial pathogens, causing a wide range of infections from mild skin infections and food poisoning to life-threatening infections, such as toxic shock syndrome, endocarditis, and osteomyelitis (1). Despite advances in antibiotic chemotherapy, including the introduction of daptomycin in 2003, treatment of S. aureus infections is still a challenge due to resistance to or tolerance of clinically used antibiotics, as exemplified by methicillin-resistant S. aureus (MRSA) or vancomycin-intermediate or -resistant S. aureus (VISA/VRSA).

S. aureus can readily acquire resistance by horizontal gene transfer (2). Without acquiring genetic mutations that actively confer antibiotic resistance, nongrowing metabolically inactive S. aureus cells also exhibit high levels of tolerance to currently used antibiotics (3–7). In a laboratory setting, the proportion of antibiotic-tolerant cells in an S. aureus population varies depending on growth phase (5, 8). In stationary phase, essentially the entire bacterial population will survive prolonged treatment with high concentrations of bactericidal antibiotics (3, 4, 6, 8). This has recently been defined as antibiotic “tolerance” (9). In contrast, in lag and early exponential phase, only a small subpopulation of an S. aureus culture will survive antibiotic treatment (8). In this case, the survivors are designated “persisters” (9).

Antibiotic tolerance is a consequence of the fact that traditional antibiotics target biosynthetic processes that are occurring at significantly reduced levels in nongrowing cells (10) or is a consequence of a low-energy state that arrests the energy-dependent uptake of antibiotics (5, 7). Clinically, bacterial tolerance or persistence is associated with the recalcitrance of chronic infections (11, 12). The present lack of effective antibiotics against antibiotic-resistant bacteria or tolerant/persister cells highlights the unmet need of developing novel antimicrobial therapies.

The validity of the bacterial membrane as an antimicrobial target has been proven by the evolution of antimicrobial peptides (AMPs) and proteins by animals, plants, and fungi that kill bacteria by disrupting bacterial membranes (13). Bacterial membranes are an ideal target for antimicrobial agents because membrane integrity is indispensable for bacterial survival regardless of growth state (14). However, a key barrier to the development of membrane-active agents as human therapeutics is their typical low level of selectivity between bacterial and mammalian membranes. Since natural AMPs exhibit high selectivity to bacterial over mammalian membranes, membrane-active small molecules are typically designed and developed to mimic natural AMPs (15, 16). Thus, rationally designed membrane-active small molecules are usually cationic and amphipathic, which are key structural features of AMPs (15, 16). In particular, the cationic nature of natural AMPs plays an important role in selectively binding to negatively charged bacterial membranes by electrostatic affinity rather than binding to zwitterionic mammalian membranes (15, 17, 18). However, bacteria can acquire resistance to cationic membrane-active agents by reducing the negative charge of their membranes, which can subsequently result in cross-resistance to a range of natural AMPs, including host innate immunity effectors and other synthetic cationic membrane-active antimicrobials (17, 18). Therefore, development of membrane-active small molecules that show high levels of bacterial membrane selectivity while at the same time minimizing the selection of cross-resistance remains challenging.

Recently, our group used a SYTOX Green-MRSA membrane permeability assay (19) to screen a collection of 185 “hits” obtained by screening ∼82,000 synthetic chemicals using an automated high-throughput Caenorhabditis elegans-MRSA intestinal infection assay (20). This strategy enables us to exclude toxic compounds with low membrane selectivity because the hit is determined based on C. elegans survival. Using this approach, we were able to identify a set of membrane-active antimicrobials effective against nongrowing MRSA. These membrane-disrupting compounds exhibited high levels of membrane selectivity to bacterial compared to mammalian membranes (6, 20, 21).

Diarylureas are known to be important pharmacophores in drug discovery (22). Indeed, diarylurea derivatives have been developed as antimalarial (23), antischistosomal (24), antimicrobial (25, 26), and anticancer (22, 27) agents. We identified the diarylurea compound PQ401 as a hit in the C. elegans screen (described above) that not only blocks the ability of MRSA to kill C. elegans but also induces rapid MRSA membrane permeabilization. PQ401 has been shown to inhibit autophosphorylation of the insulin-like growth factor I receptor (IGF-1R) and impede breast cancer cell growth in *in vivo* mouse models (28). However, the antimicrobial activity of PQ401 has not been previously reported. In this paper, we elucidate the mode of action by which PQ401 permeabilizes the membrane and the role that ionized states of PQ401 play in its antimicrobial potency. Unexpectedly, we found that the neutral form of PQ401 is more potent than the cationic form, which correlates with enhanced membrane penetration of the neutral form in molecular dynamics simulation studies. In addition, we report that PQ401 has promising features as a potential therapeutic including high potency against both multidrug-resistant and multidrug-tolerant Gram-positive pathogens, fast killing kinetics, a very low rate of resistance development, and synergism with gentamicin.

## RESULTS

### PQ401 exhibits bactericidal activity and a low probability for resistance development.

**FIG 1:**
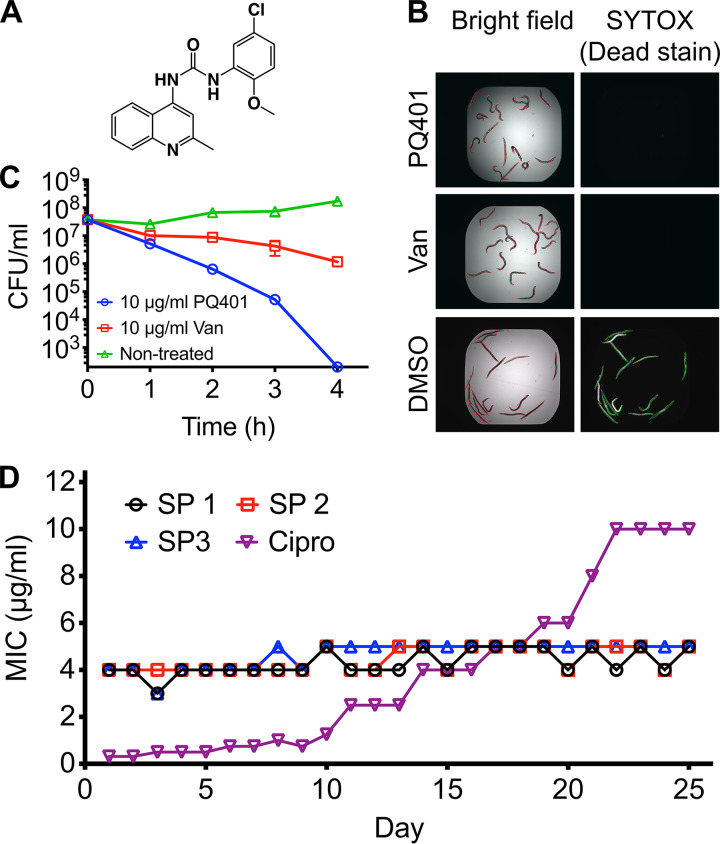
The insulin-like growth factor receptor (IGF-1R) inhibitor PQ401 exhibits bactericidal activity against S. aureus strain MW2 with no detectable resistance development. (A) Chemical structure of PQ401. (B) MRSA-infected C. elegans was treated with 5 μg/ml PQ401, 10 μg/ml vancomycin (Van, positive control), and 0.1% DMSO (DMSO, negative control). Dead worms were stained with SYTOX Orange. (C) Exponential-phase S. aureus MW2 was treated with PQ401 or vancomycin for 4 h. The bacterial viability was measured at hourly intervals. The limitation of detection is 2 × 10^2^ CFU/ml. Error bars denote SD (*n* = 3). (D) Three attempts to develop MRSA resistance to PQ401 (SP 1, 2, and 3) and to ciprofloxacin (Cipro) over 25 days.

We identified PQ401 (Fig. 1A) as a hit compound that rescues C. elegans from MRSA-mediated killing (Fig. 1B) (20). In general, a compound can rescue C. elegans from an MRSA intestinal infection if it inhibits bacterial growth, blocks a bacterial virulence factor or factors, or modulates C. elegans innate immunity (29–31). We first tested the antimicrobial activity of PQ401 against a panel of antibiotic-resistant S. aureus strains, including MRSA clinical isolates and a vancomycin-resistant S. aureus (VRSA; strain VRS1) (32). As shown in Table 1, the MIC of PQ401 was 4 μg/ml against all of the MRSA strains tested as well as the VRSA strain VRS1 (Table 1). PQ401 demonstrated bactericidal activity with a minimum bactericidal concentration (MBC) of 4 μg/ml against a panel of MRSA and VRSA strains (Table 2). It exhibited fast killing kinetics, completely eradicating 5 × 10^7^ CFU/ml of growing MRSA at 10 μg/ml within 4 h, indicating that PQ401 is a more effective bactericidal agent than vancomycin against MRSA (Fig. 1C).

**TABLE 1:** MIC of PQ401 against MRSA strains

MRSA strain	MIC (μg/ml) of drug:		
	Oxacillin	Vancomycin	PQ401
MW2	64	1	4
ATCC 33591	>64	2	4
JE2	64	1	4
VRS1	>64	>64	4
BF1	>64	2	4
BF2	>64	2	4
BF3	32	2	4
BF4	16	2	4
BF5	>64	1	4
BF7	>64	2	4
BF8	>64	2	4
BF10	>64	1	4
BF11	>64	1	4

**TABLE 2:** Minimum bactericidal concentration of PQ401 against MRSA strains

MRSA strain	MBC (μg/ml) of drug:		
	Oxacillin	Vancomycin	PQ401
MW2	>64	4	4
ATCC 33591	>64	4	4
JE2	>64	8	4
VRS1	>64	>64	4

We further tested the antimicrobial activity of PQ401 using a panel of the so-called ESKAPE pathogens, which include two Gram-positive bacteria (Enterococcus faecium and S. aureus) and four Gram-negative bacteria (Klebsiella pneumoniae, Acinetobacter baumannii, Pseudomonas aeruginosa, and *Enterobacter* spp.). These ESKAPE pathogens are of particular concern because they are the leading cause of nosocomial infections and often develop antibiotic resistance (33). PQ401 showed antimicrobial activity against Gram-positive pathogens but not against Gram-negative pathogens (Table 3). Interestingly, PQ401 has an MIC of 4 μg/ml against the multidrug-resistant E. faecium strain C68 (34) (Table 3) as it does against the S. aureus strains tested.

**TABLE 3:** MICs of PQ401 against the ESKAPE pathogens

Strain	MIC (μg/ml) of drug:			
	PQ401	Vancomycin	Gentamicin	Ciprofloxacin
*Enterococcus faecium* E007	4	1	>64	>64
*Enterococcus faecium* C68	4	64	>64	>64
*Staphylococcus aureus* Newman	4	2	2	0.25
*Klebsiella pneumoniae* WGLW2	>64	>64	1	0.031
*Acinetobacter baumannii* ATCC 17978	>64	>64	1	0.25
*Pseudomonas aeruginosa* PA14	>64	>64	2	0.063
*Enterobacter aerogenes* ATCC 13048	>64	>64	2	0.031

S. aureus can readily develop resistance against most clinical antibacterial agents (35). To evaluate the ability of S. aureus to develop resistance to PQ401, we exposed three independent cultures of MRSA strain MW2 (SP1, SP2, and SP3) to a sub-MIC level of PQ401 for 25 days using a serial passage method in a 96-well plate (36). The fluoroquinolone antibiotic ciprofloxacin targeting DNA gyrase was used as a control. MRSA MW2 strains exhibiting a 32-fold-higher MIC to ciprofloxacin than the wild-type strain were generated after 25 days of serial passage in sub-MICs (Fig. 1D). In contrast, we did not observe a significant increase in the PQ401 MIC during the same time frame (Fig. 1D). This result suggests that PQ401 exhibits an extremely low probability for PQ401 resistance development.

### PQ401 selectively disrupts bacterial membranes.

**FIG 2:**
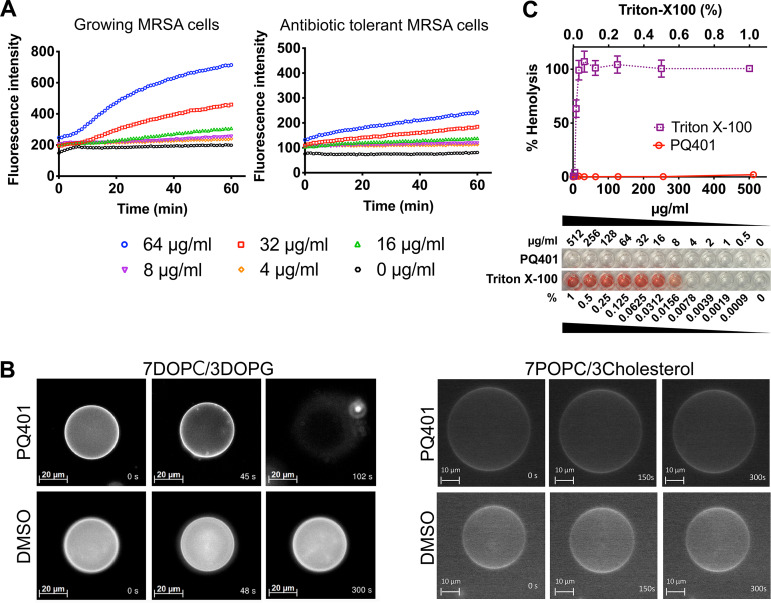
PQ401 selectively disrupts MRSA membranes. (A) Elicitation of membrane permeability by PQ401 for growing MRSA cells or stationary-phase antibiotic-tolerant MRSA cells. Membrane permeability was evaluated by monitoring the uptake of the membrane-impermeable dye SYTOX Green for 60 min. Results are shown as means from triplicates. Error bars (SD) are excluded for clarity. (B) GUVs consisting of DOPC/DOPG (7:3) or POPC/cholesterol (7:3) labeled with 0.05% Liss Rhod PE were treated with 4 μg/ml PQ401 or 0.1% DMSO. Deformation dynamics of GUVs was monitored over time using fluorescence microscopy. (C) Two percent human erythrocytes were treated with a range of PQ401 concentrations for 1 h at 37°C. A sample treated with 1% Triton X-100, which causes 100% hemolysis, was used as the positive control. Results are shown as means ± SD (*n* = 3).

The finding that PQ401 exhibits a high rate of killing and a low probability of resistance development suggests that it might be functioning as a membrane-active antimicrobial (14, 37). To test whether PQ401 disrupts MRSA membranes, we measured the permeability of MRSA MW2 to SYTOX Green after treatment with a range of concentrations of PQ401. As shown in Fig. 2A, PQ401 induced rapid membrane permeabilization in a dose-dependent manner. Dose-dependent permeabilization was also observed in nongrowing antibiotic-tolerant MRSA cells treated with PQ401. These results indicate that PQ401 can cause membrane damage regardless of growth states.

To further explore the effects of PQ401 on bacterial lipid bilayers, we challenged biomembrane-mimicking giant unilamellar vesicles (GUVs) with PQ401. GUVs are artificial spherical vesicles made up of a single lipid bilayer with a diameter of 10 to 100 μm (38, 39). Their relatively large size enables direct observation of dynamic morphological changes by optical microscopy (40, 41). GUVs have been employed to investigate the modes of action of several membrane-active antibacterial agents, including daptomycin (38, 42–45). To mimic the negatively charged S. aureus membrane, we created GUVs consisting of a dioleoyl-*sn*-glycero-3-phosphocholine (DOPC)/1,2-dioleoyl-*sn*-glycero-3-phospho-(1′-*rac*-glycerol) (DOPG) lipid bilayer at a ratio of 7:3, which have been used for monitoring the effects of daptomycin and other membrane-active antimicrobial agents on S. aureus lipid bilayers (38, 46–49). When GUVs were treated with 4 μg/ml (1× MIC) PQ401, we noted the formation of lipid aggregates on the surface of the GUVs after ∼45 s, followed by rupture at ∼100 s (Fig. 2B; see also Movies S1 and S2 in the supplemental material), indicating that PQ401 directly interacts with and disrupts bacterial mimetic lipid bilayers.

10.1128/mBio.01140-20.2 MOVIE S1 GUVs consisting of DOPC/DOPG (7:3) labeled with 18:1 Liss Rhod PE (0.05%) were treated with 4 μg/ml PQ401. After adding compounds at *t* = 0 s, GUVs were monitored using a fluorescent microscope (63× objective, excitation = 460 nm, emission = 483 nm). Download Movie S1, MOV file, 2.1 MB. Copyright © 2020 Kim et al. 2020 Kim et al. This content is distributed under the terms of the Creative Commons Attribution 4.0 International license.

10.1128/mBio.01140-20.3 MOVIE S2 GUVs consisting of DOPC/DOPG (7:3) labeled with 18:1 Liss Rhod PE (0.05%) were treated with 0.1% DMSO. After adding compounds at *t* = 0 s, GUVs were monitored using a fluorescent microscope (63× objective, excitation = 460 nm, emission = 483 nm). Download Movie S2, MOV file, 7.1 MB. Copyright © 2020 Kim et al. 2020 Kim et al. This content is distributed under the terms of the Creative Commons Attribution 4.0 International license.

Membrane-active agents typically interact with both bacterial and mammalian lipid bilayers. To test the membrane selectivity of PQ401, we fabricated GUVs consisting of 1-palmitoyl-2-oleoyl-glycero-3-phosphocholine (POPC) and cholesterol in the ratio of 7:3, which mimics mammalian lipid bilayers (50, 51). In contrast to the bacterial mimetic GUVs, PQ401 did not induce any deformation of the mammalian mimetic GUVs at 4 μg/ml or 10 μg/ml (Fig. 2B and Movies S3 to S5). The inertness of mammalian membranes to PQ401 was confirmed using human erythrocytes. As shown in Fig. 2C, PQ401 did not induce detectable hemolysis of erythrocytes up to 512 μg/ml. Consistent with these results, PQ401 was previously reported to kill cancer cells by IGF-1R inhibition-mediated apoptosis rather than membrane disruption (28, 52). Combined, these results indicate that PQ401 has a high level of selectivity for bacterial in comparison to mammalian membranes.

10.1128/mBio.01140-20.4 MOVIE S3 GUVs consisting of POPC/cholesterol (7:3) labeled with 18:1 Liss Rhod PE (0.05%) were treated with 4 μg/ml PQ401. After adding compounds at *t* = 0 s, GUVs were monitored using a fluorescent microscope (63× objective, excitation = 460 nm, emission = 483 nm). Download Movie S3, MOV file, 1.8 MB. Copyright © 2020 Kim et al. 2020 Kim et al. This content is distributed under the terms of the Creative Commons Attribution 4.0 International license.

### Neutral PQ401 penetrates into bacterial lipid bilayers.

**FIG 3:**
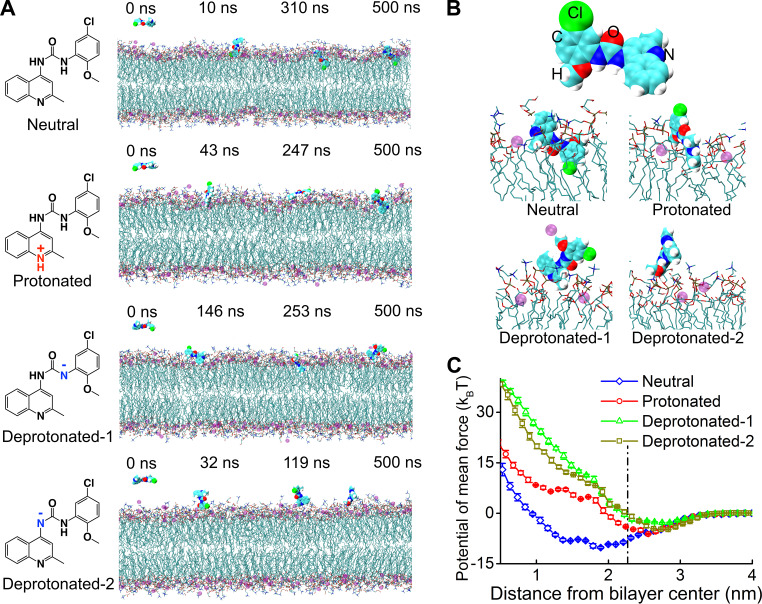
Only the neutral form of PQ401 is predicted to penetrate into bacterial lipid bilayers. (A) Representative simulated configurations of PQ401 in different ionized states from left to right: onset, membrane attachment, membrane penetration, and equilibrium interacting with 7DOPC/3DOPG lipid bilayers. PQ401 and sodium ions are depicted as large spheres; phospholipids are represented as chains. The atoms in PQ401, phospholipids, and sodium ions are colored as follows: hydrogen, white; oxygen, red; nitrogen, blue; chlorine, green; carbon, cyan; phosphorus, orange; sodium, purple. Water molecules are not shown for clarity. (B) Magnified view of PQ401 and the corresponding simulated configurations at 500 ns in different ionized states. (C) The free energy profiles of PQ401 in different ionized states penetrating into the lipid bilayer as a function of the center-of-mass (COM) distance to the bilayer. The dot-dashed black line marks the surface of the membrane, averaged from the COM locations of phosphate groups in the lipids of the outer leaflet. Error bars represent means ± SD from three independent simulations.

To further explore the molecular details by which PQ401 interacts with bacterial membranes, we conducted all-atom molecular dynamics (MD) simulations. The topology and parameters of PQ401 for the GROMOS54a7 forcefield (53) were generated by Automated Topology Builder (ATB) (54, 55). Like bacterial membrane-mimetic GUVs, we used the previously established model of DOPC/DOPG at a 7:3 ratio to simulate negatively charged S. aureus membranes. The MD modeling showed that PQ401 is initially recruited to the membrane surface by the binding of the chloro-methoxyphenyl moiety to hydrophilic lipid heads via the polar interactions between two polar moieties, including the urea and chloro-methoxyphenyl groups and hydrophilic lipid head groups (Fig. 3A; see also Fig. S1A and Movie S6). After several tens of nanoseconds of sustained attachment, PQ401 penetrates into the membrane interior, maximizing interactions between a nonpolar benzene ring and hydrophobic lipid tails (Fig. 3A and B and Movie S6). Potential mean force (PMF) calculations (Fig. S1B) using the umbrella sampling method (56) confirmed that insertion of PQ401 into the lipid bilayer is energetically favorable with a transfer energy about −10 *k_B_*T (Fig. 3C).

10.1128/mBio.01140-20.1 FIG S1 (A) Time evolutions of the distance, the interaction energy, and the corresponding Van der Waals and Coulomb interaction energy between PQ401 in different ionized states and the bacterial membrane in MD simulations. (B) Selected snapshots from umbrella simulations with a reference distance about 3.0 nm, 2.2 nm, 1.5 nm, and 1.0 nm and the histograms of the umbrella sampling. (C) Free-energy profiles of PQ401 penetrating into the indicated lipid bilayers as a function of the center-of-mass (COM) distance to the bilayer and the corresponding histogram of umbrella sampling. The dot-dashed lines mark the surface of membrane, averaged from the COM locations of phosphate groups in the lipids of the outer leaflet. Download FIG S1, PDF file, 1.5 MB. Copyright © 2020 Kim et al. 2020 Kim et al. This content is distributed under the terms of the Creative Commons Attribution 4.0 International license.

10.1128/mBio.01140-20.5 MOVIE S4 GUVs consisting of POPC/cholesterol (7:3) labeled with 18:1 Liss Rhod PE (0.05%) were treated with 10 μg/ml PQ401. After adding compounds at *t* = 0 s, GUVs were monitored using a fluorescent microscope (63× objective, excitation = 460 nm, emission = 483 nm). Download Movie S4, MOV file, 0.9 MB. Copyright © 2020 Kim et al. 2020 Kim et al. This content is distributed under the terms of the Creative Commons Attribution 4.0 International license.

10.1128/mBio.01140-20.6 MOVIE S5 GUVs consisting of POPC/cholesterol (7:3) labeled with 18:1 Liss Rhod PE (0.05%) were treated with 0.1% DMSO. After adding compounds at *t* = 0 s, GUVs were monitored using a fluorescent microscope (63× objective, excitation = 460 nm, emission = 483 nm). Download Movie S5, MOV file, 1.8 MB. Copyright © 2020 Kim et al. 2020 Kim et al. This content is distributed under the terms of the Creative Commons Attribution 4.0 International license.

10.1128/mBio.01140-20.7 MOVIE S6 Molecular dynamics simulation of neutral PQ401 interacting with a 7DOPC:3DOPG lipid bilayer. Neutral PQ401 and sodium ions are depicted as large spheres, and phospholipids are represented as chains. The atoms in PQ401 phospholipids and sodium ions are colored as follows: hydrogen, white; oxygen, red; nitrogen, blue; chlorine, green; carbon, cyan; phosphorus, orange; sodium, blue. Water molecules are not shown for clarity. The outer blue lines indicate the periodic boundaries of the simulation boxes. Download Movie S6, MOV file, 3.0 MB. Copyright © 2020 Kim et al. 2020 Kim et al. This content is distributed under the terms of the Creative Commons Attribution 4.0 International license.

Next, we also conducted MD simulations to explore PQ401 interaction with the mammalian mimetic lipid bilayer of POPC-cholesterol at a 7:3 ratio. Unexpectedly, we found that the MD simulations predicted that PQ401 could also penetrate into the mammalian membrane with a slightly higher transfer energy than with the bacterial mimetic membrane (Fig. S1C). Although this simulation indicates that the penetration of PQ401 into mammalian lipid bilayers is energetically favorable, as described above, human red blood cells as well as GUVs having the same composition of POPC-cholesterol as the membranes used in the MD simulations (Fig. 2B and C and Movies S3 to S5) are resistant to disruption by PQ401. Combined, these data indicate that the penetration of PQ401 molecules *per se* is not sufficient to induce the disruption of mammalian lipid bilayers.

The ionization states of PQ401 can be varied. The MarvinSketch program (ChemAxon Ltd.) predicts that PQ401 has 4 different ionization states: a neutral, a protonated, and two deprotonated forms at the ratios of 98.48 to 1.45 to 0.06 to 0.02, respectively, at pH 7.4 (Fig. 3A; Table 4). To address whether the ionization state affects membrane activity, we conducted additional MD simulations. In contrast to the neutral form, the penetration of all three of the ionized forms into lipid bilayers was not energetically favorable (Fig. 3C). The mechanisms by which each of the ionized forms interacts with lipid bilayers are somewhat different. In the case of the protonated form, unlike the neutral form, the positively charged methylquinoline group instead of the chloro-methoxyphenyl moiety drives the molecule to the negatively charged surface of the lipid bilayer; however, the strong binding between the positively charged methylquinoline group and the negatively charged membrane surface prevents further penetration (Fig. 3A and B, Fig. S1A, and Movie S7). In the case of two deprotonated forms, like the neutral form, the chloro-methoxyphenyl moiety binds on the surface of the bilayer membrane; however, it is unable to penetrate into lipid bilayers because of the electrostatic repulsion between the negatively charged nitrogen and negatively charged lipid head groups (Fig. 3A and B, Fig. S1B, and Movies S8 and S9).

**TABLE 4:** Proportion of each ionized form of PQ401 and their MICs against MRSA strain MW2^*a*^

pH	% ratio (N:P:Dp1:Dp2)	MIC (μg/ml) of drug:	
		PQ401	Vancomycin
5.5	46.15:53.85:0.00:0.00	16	1
6.5	89.54:10.45:0.01:0.00	4	1
7.4	98.48:1.45:0.06:0.02	4	1
8.5	98.96:0.12:0.72:0.02	4	1

a Abbreviations: N, neutral form; P, protonated form; Dp1, deprotonated-1 form; Dp2, deprotonated-2 form shown in Fig. 3. Ionized states were estimated by MarvinSketch.

10.1128/mBio.01140-20.8 MOVIE S7 Molecular dynamics simulation of protonated PQ401 interacting with a 7DOPC:3DOPG lipid bilayer. Protonated PQ401 and sodium ions are depicted as large spheres, and phospholipids are represented as chains. The atoms in PQ401 and phospholipids and sodium ions are colored as follows: hydrogen, white; oxygen, red; nitrogen, blue; chlorine, green; carbon, cyan; phosphorus, orange; sodium, blue. Water molecules are not shown for clarity. The outer blue lines indicate the periodic boundaries of the simulation boxes. Download Movie S7, MOV file, 3.0 MB. Copyright © 2020 Kim et al. 2020 Kim et al. This content is distributed under the terms of the Creative Commons Attribution 4.0 International license.

10.1128/mBio.01140-20.9 MOVIE S8 Molecular dynamics simulation of the first deprotonated PQ401 interacting with a 7DOPC:3DOPG lipid bilayer. Deprotonated PQ401 and sodium ions are depicted as large spheres, and phospholipids are represented as chains. The atoms in PQ401, phospholipids, and sodium ions are colored as follows: hydrogen, white; oxygen, red; nitrogen, blue; chlorine, green; carbon, cyan; phosphorus, orange, sodium, blue. Water molecules are not shown for clarity. The outer blue lines indicate the periodic boundaries of the simulation boxes. Download Movie S8, MOV file, 3.0 MB. Copyright © 2020 Kim et al. 2020 Kim et al. This content is distributed under the terms of the Creative Commons Attribution 4.0 International license.

10.1128/mBio.01140-20.10 MOVIE S9 Molecular dynamics simulation of the second deprotonated PQ401 interacting with a 7DOPC:3DOPG lipid bilayer. Deprotonated PQ401 and sodium ions are depicted as large spheres, and phospholipids are represented as chains. The atoms in PQ401, phospholipids, and sodium ions are colored as follows: hydrogen, white; oxygen, red; nitrogen, blue; sulfur, yellow; chlorine, green; carbon, cyan; phosphorus, orange; sodium, blue. Water molecules are not shown for clarity. The outer blue lines indicate the periodic boundaries of the simulation boxes. Download Movie S9, MOV file, 3.0 MB. Copyright © 2020 Kim et al. 2020 Kim et al. This content is distributed under the terms of the Creative Commons Attribution 4.0 International license.

**FIG 4:**
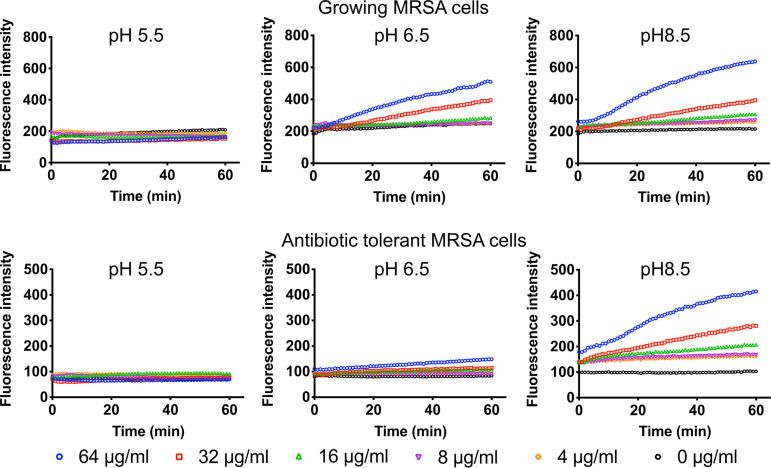
The membrane permeability of PQ401 is augmented as pH increases. Membrane permeability by PQ401 at the indicated pH was evaluated by monitoring the uptake of SYTOX Green for 60 min. Results are shown as means from triplicates. Error bars (SD) are excluded for clarity.

To verify these MD simulation results experimentally, we decreased the portion of the neutral PQ401 by lowering the pH. The MarvinSketch program predicts that at pH 5.5, more than 50% of PQ401 exists in a protonated form, which, according to the MD simulations, is unable to penetrate the membrane (Table 4 and Fig. 3). Consistent with the MD simulations, the MIC of PQ401 at pH 5.5 increased to 16 μg/ml, which is 4-fold higher than at pH 7.4 (Table 4). Further, the ability of PQ401 to permeabilize the membrane of both growing and nongrowing antibiotic-tolerant MRSA was significantly decreased at pH 5.5 (Fig. 4). As pH increased, the membrane permeability of PQ401 also increased (Fig. 4). Between pH 6.5 and 8.5, the MIC of PQ401 was 4 μg/ml, where the neutral portion of PQ401 is 90% or greater. These computational and experimental results demonstrate that the polarity of branch groups, the hydrophobicity of core rings, and the ionization state play important roles in the membrane activity of PQ401.

### PQ401 kills antibiotic-tolerant MRSA and shows synergism with gentamicin.

**FIG 5:**
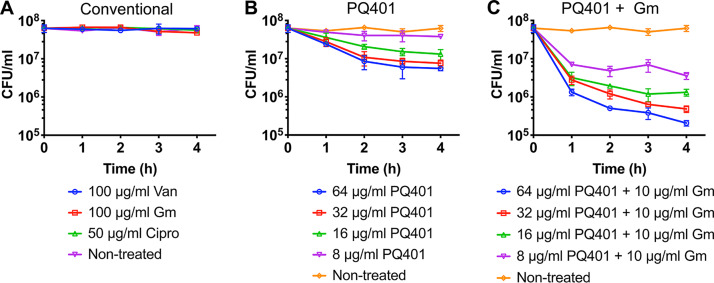
PQ401 has bactericidal potency and shows synergism with gentamicin against antibiotic-tolerant MRSA. Antibiotic-tolerant cells of MRSA MW2 were treated with 100× MIC vancomycin (Van), gentamicin (Gm), or ciprofloxacin (Cipro) (A); the indicated concentrations of PQ401 (B); or 10 μg/ml gentamicin (Gm) in combination with various concentrations of PQ401 for 4 h (C). CFU counts of viable cells were measured by serial dilution and plating on TSA plates. Results are shown as means ± SD (*n* = 3).

As shown above, PQ401 induces membrane permeabilization of antibiotic-tolerant MRSA cells (Fig. 2A). Thus, we reasoned that PQ401 should be effective against antibiotic-tolerant MRSA. As previously reported and shown here in Fig. 5A, 100% of stationary-phase MRSA cells become antibiotic-tolerant cells that are not susceptible to a panel of antibiotics having different modes of action. Indeed, PQ401 showed bactericidal activity against antibiotic-tolerant MRSA cells in a dose-dependent manner, albeit with significantly less activity than against growing MRSA. PQ401 caused a 1-log reduction in antibiotic-tolerant MRSA viability at 64 μg/ml (4× MIC) (Fig. 5B).

Membrane-active antimicrobial agents are known to act synergistically with aminoglycosides against antibiotic-tolerant bacteria by facilitating the diffusion of the aminoglycoside across bacterial membranes (6, 20, 21, 57). We tested the synergism of PQ401 with the aminoglycoside antibiotic gentamicin against antibiotic-tolerant MRSA cells. As shown in Fig. 5C, combined with gentamicin, the bactericidal activity of PQ401 was enhanced against antibiotic-tolerant MRSA. The combination of 64 μg/ml PQ401 and 10 μg/ml gentamicin led to a ∼3-log reduction in antibiotic-tolerant MRSA viability (Fig. 5C).

### PQ401 is efficacious in invertebrate animal models.

**FIG 6:**
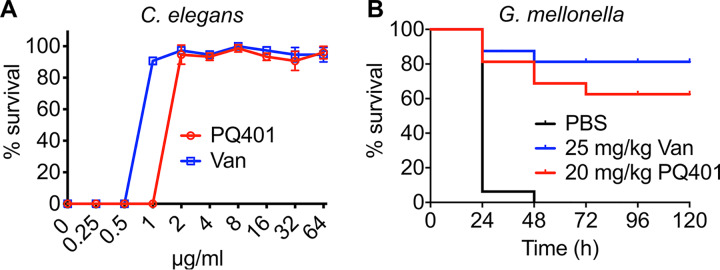
PQ401 shows efficacy in two invertebrate animal infection models. (A) MRSA-infected C. elegans *glp-4(bn2);sek-1(km4)* animals were treated with the indicated concentrations of PQ401 or vancomycin at 25°C for 5 days. After staining dead worms with SYTOX Orange, percent survival of C. elegans was calculated in each well of the assay plate. Results are shown as means ± SD (*n* = 3). (B) Sixteen MRSA-infected G. mellonella larvae (*n *= 16) were treated with control (PBS), 25 mg/kg vancomycin (Van), or 20 mg/kg PQ401 at 1 h postinfection. Larval survival following treatment with 20 mg/kg PQ401 was significant compared to PBS treatment (*P < *0.0001). Data are representative of two independent experiments.

To test the efficacy of PQ401, we used two invertebrate model hosts, C. elegans and Galleria mellonella (wax moth) larvae. These two infection models are widely used to evaluate *in vivo* antimicrobial potential and toxicity, which can fill the gap between *in vitro* and *in vivo* mammalian experiments (29, 30, 58). In a C. elegans-MRSA infection model, PQ401 exhibited a median effective concentration (EC_50_) of 1.7 μg/ml, which is ∼2-fold higher than the EC_50_ (0.86 μg/ml) of vancomycin (Fig. 6A). PQ401 completely blocked C. elegans death from MRSA infection at ∼2 μg/ml, which, interestingly, is ∼2-fold lower than its MIC of 4 μg/ml, whereas vancomycin provided 100% worm survival at around the MIC. In addition, the exposure of MRSA-infected C. elegans at 64 μg/ml PQ401 for 5 days did not affect C. elegans viability (Fig. 6A).

Next, we tested the efficacy of PQ401 in G. mellonella larvae. All MRSA-infected G. mellonella larvae were dead within 48 h postinfection. We treated the infected larvae with 20 mg PQ401/kg of body weight by injecting 10 μl of 0.5 mg/ml PQ401, which is the maximum injectable dose due to its solubility in phosphate-buffered saline (PBS). Also, PQ401 does not cause hemolysis at this concentration of 0.5 mg/ml (Fig. 2C). The PQ401-treated larvae showed 62.5% survival at 120 h postinfection (Fig. 6B), indicating significant efficacy (*P < *0.0001, Fig. 6B). Consistent with MIC and EC_50_ results (Table 1 and Fig. 6A), PQ401 efficacy was lower than vancomycin, showing 81.5% survival at 120 h postinfection (Fig. 6B). Taken together, PQ401 is significantly efficacious in the two MRSA infection animal models.

## DISCUSSION

Major disadvantages of conventional antibiotics include resistance development and inactivity against nongrowing antibiotic-tolerant bacteria. Ideally, the targets of a new generation of antibiotics should be growth independent and pathogens should exhibit very low rates of resistance development to antimicrobials corresponding to these targets. Membrane-disruptive antimicrobial agents have a potential to overcome the drawbacks of conventional antibiotics if they exhibit membrane selectivity to bacterial compared to mammalian membranes (14, 59). PQ401 is a new example of a class of membrane-active agents that we have recently described that exhibit antimicrobial potency against multidrug-resistant and multidrug-tolerant S. aureus, low probability for the development of resistance, and the ability to act synergistically with other antibiotics as well as high membrane selectivity to Gram-positive bacterial membranes.

Interestingly, PQ401 rescued 100% of C. elegans worms from MRSA infections at one-half the MIC, whereas vancomycin rescued 100% only at 1× MIC (Fig. 6A), indicating that PQ401 may provide additional beneficial bioactivity to combat MRSA infections. PQ401 may suppress the expression of MRSA virulence at subinhibitory concentrations. Several antibiotics, such as linezolid and tigecycline, have been shown to reduce S. aureus virulence factor expression at sub-MIC levels (60). Also, our laboratory reported previously that a different membrane-active antimicrobial, NH125, downregulated the expression of several virulence factors, including alpha-hemolysin, delta-hemolysin, coagulase, and nuclease, at subinhibitory concentrations (61). At sub-MIC levels, antibacterial compounds targeting protein synthesis may decrease virulence gene expression (60), and subinhibitory concentrations may also act as a selective pressure for resistant development (62). However, since PQ401 displays a very low probability for resistance development at sub-MIC levels, it is possible that its antivirulence activity at subinhibitory concentrations could contribute to its ability to treat MRSA infections.

The design and development of membrane-active small molecules have been traditionally based on a strategy that involves mimicking natural AMPs secreted from a variety of host organisms, including both plant and animal species, because their membrane selectivity and antimicrobial activity have been optimized through evolution in nature (63, 64). In general, common features of AMPs include a net charge of ∼+3 and a hydrophobic content of ∼42% (15, 65, 66). Arginine and lysine are responsible for the cationic characteristics; tryptophan, phenylalanine, leucine, and isoleucine contribute to hydrophobicity (63). In particular, the cationic nature of AMPs plays a key role in selective binding to negatively charged bacterial membranes rather than to zwitterionic mammalian membranes (15, 63). Following an initial electrostatic interaction with a bacterial membrane, the hydrophobic residues of AMPs interact with bacterial lipid tails (15, 63). Based on this paradigm, rationally designed membrane-active small molecules usually exhibit cationic and amphipathic structures (15, 16) and have a similar mode of action as AMPs. Interestingly, although daptomycin, an FDA-approved membrane-active and last-resort antibacterial against MRSA, is anionic, it forms a cationic complex with Ca^2+^ and therefore uses electrostatic attraction to interact with negatively charged bacterial membranes similarly to AMPs (67).

A disadvantage of cationic AMPs and cationic low-molecular-weight compounds as antimicrobial therapeutics is that the electrostatic binding of cationic antimicrobials to anionic bacterial membranes can result in the selection of AMP-resistant bacteria that exhibit an overall reduced negative charge (17, 18). Such mutants often exhibit cross-resistance to a broad spectrum of cationic membrane-active antimicrobials, which includes daptomycin (18). Moreover, AMP-resistant bacteria are potentially also more resistant to host innate immunity-related effectors such as defensins and cathelicidins that target bacterial membranes (18). For example, daptomycin-resistant S. aureus strains exhibit cross-resistance to host defense peptides including human neutrophil defensin-1 and LL-37 (68, 69).

In contrast to conventional membrane-active antimicrobial small molecules and peptides, the membrane activity of PQ401 is maximized when it exists in a neutral form rather than a cationic form (Fig. 3). Furthermore, PQ401 does not have clear lipophilic elements, such as acyl chains. In the case of PQ401, two polar moieties, the chloro-methoxyphenyl and urea groups, provide sufficient affinity to bind PQ401 to the surface of the lipid bilayer. Importantly, this attraction of the chloro-methoxyphenyl group to the membrane surface is not so strong that it hinders further penetration of PQ401 and subsequent interaction with hydrophobic lipid tails (Fig. 3; see also Movie S6 in the supplemental material). In light of the development of cross-resistance to cationic membrane-active compounds, neutral antimicrobial membrane-targeting compounds such as PQ401 should circumvent this issue.

To date, we have described four different classes of small-molecule membrane-active agents including NH125 (19, 49), synthetic retinoids (CD437, CD1530) (20), nTZDpa (21), and bithionol (6) that are effective against antibiotic-tolerant MRSA cells. Except for NH125, which is cationic and amphipathic, the remaining three compounds, as well as PQ401, are neither cationic nor amphipathic but also exhibit significant differences from each other. CD437 and nTZDpa have a carboxylic moiety and predominately exist as a negatively charged deprotonated form at pH 7.4. According to predictions made using MarvinSketch, more than 50% of bithionol also exists as anionic forms at pH 7.4. Previously, our laboratory reported MD simulations which show that the neutral forms of CD437, nTZDpa, and bithionol penetrate into lipid bilayers (6, 20, 21). Our working hypothesis is that their anionic forms probably have significantly reduced membrane activity due to electrostatic repulsion to negatively charged bacterial membranes as shown in the case of anionic forms of PQ401 (Fig. 3).

Unlike PQ401, however, only relatively small portions of the neutral forms of CD437, nTZDpa, and bithionol exist at pH 7.4. The reason why CD437, nTZDpa, and bithionol are such effective membrane disrupters at neutral pH is not understood, but one possibility is that the neutral forms of these molecules accumulate over time in bacterial membranes, ultimately reaching high-enough concentrations to disrupt membrane function. From a kinetic point of view, embedment of these neutral molecules into bacterial membranes is very fast (within hundreds of nanoseconds), energetically favorable, and almost irreversible (Fig. 3A and C and Movie S6) (6, 20, 21). Once embedded, the energy cost (∼10 *k_B_*T) to remove these molecules from the membranes is high (Fig. 3C) (6, 20, 21). Further, at physiological pH, the ratio of anionic and neutral forms of these molecules in solution remains constant to satisfy the Henderson-Hasselbach equation (pH = pK_a_ + log_10_ [A-]/[HA]). Thus, because only a small portion of these molecules are present in a neutral form at physiological pH, as neutral forms are embedded into the membranes, more neutral forms are generated from anionic forms to essentially maintain a constant concentration of neutral molecules outside the bacteria (70). In any case, cationic forms of CD437, nTZDpa, or bithionol do not exist at any physiological pH. Therefore, CD437, nTZDpa, and bithionol as well as PQ401 demonstrate that unconventional small molecules can bind to and disrupt negatively charged bacterial membranes in a cationic-independent manner.

Although PQ401 did not cause either disruption of the mammalian mimetic GUVs or lysis of human red blood cells at concentrations up to 512 μg/ml (Fig. 2B and C and Movies S3 to S5), the MD simulations predicted that PQ401 could penetrate into simulated mammalian lipid bilayers (Fig. S1C), suggesting that there is a discrepancy between the MD simulation and experimental results. However, it is important to point out that the all-atom MD simulations evaluate only the ability of a single molecule to penetrate a lipid bilayer based on free energy profiles. In contrast, our previous studies showed that at least three factors including a molecule’s ability to attach to, penetrate, and perturb membrane lipid bilayers play important roles in membrane disruption (6, 20, 21). Thus, our current data with PQ401 suggest that it is possible that the collective behavior of a group of molecules rather than the behavior of single molecules is required for the disruption of lipid bilayers. For example, groups of interacting daptomycin molecules form oligomeric pores in bacterial lipid bilayers that induce membrane disruption (67). Moreover, the physical properties of particular lipid bilayers confer different levels of resistance to disruption to membrane-penetrating compounds. The 7POPC/3Cholesterol bilayer is much stiffer (over 100 *k_B_*T) than the 7DOPC/3DOPG (∼20 *k_B_*T) bilayer (71). Therefore, it is not unexpected that simulations of the interaction between a single molecule and a lipid bilayer do not reflect all of the features observed in our wet-lab experiments with GUVs and red blood cells.

Despite the attractive properties of PQ401 as a potential lead compound for the development of an antimicrobial therapeutic, PQ401 has limitations that require further development. First, PQ401 was identified as an inhibitor of IGF-1R that induces the apoptosis of a variety of cancer cells by blocking the autophosphorylation of IGF-1R (28, 52). IGF-1R plays important roles in several cellular processes, including cell proliferation, development, and survival. Dysregulation of IGF-1R results in many diseases, including cancers, thyroid eye disease, psoriasis, and diabetes (72). Therefore, for the further development of PQ401 as an antimicrobial therapeutic, it would be critical to minimize potential toxic side effects of PQ401 by nullifying its IGF-1R-inhibitory activity while maintaining its antimicrobial activity. Second, the MIC of PQ401 is 4 μg/ml against proliferating MRSA MW2 cells (Table 1), which is 4-fold higher than the MIC (∼1 μg/ml) of vancomycin, daptomycin, and linezolid (6, 20), which are currently used to treat MRSA infections. Moreover, PQ401 treatment of nongrowing antibiotic-tolerant MRSA at 64 μg/ml resulted in only a 10-fold decrease in viability (Fig. 5B), whereas other membrane-active antimicrobials recently identified in our laboratory, such as particular synthetic retinoids, nTZDpa, and bithionol, completely eradicated antibiotic-tolerant MRSA at this concentration (6, 20, 21). Finally, PQ401 exhibits poor aqueous solubility. In the case of the G. mellonella larva infection model, 20 mg/kg was the maximum dose possible.

Fortunately, however, it seems likely that it should be feasible to eliminate the IGF-1R-inhibitory activity of PQ401 as well as improve its antimicrobial activity and aqueous solubility and decrease its toxicity by appropriate structural modifications. First of all, the crystal structure of IGF-1R has been determined (73). Based on this structural information, the interactions between IGF-1R and small-molecule inhibitors are well defined, and extensive *in silico* molecular docking analyses have been carried out (74–76). Combining what is known about the well-defined interaction mechanism between small molecules and IGF-1R with our data pertinent to the penetration of PQ401 into Gram-positive bacterial lipid bilayers, it seems likely that it would be possible to eliminate the IGF-1R-inhibitory activity of PQ401 without the loss of its bacterial membrane-disrupting activity. In this regard, it is noteworthy that replacement of a carboxyl acid with a primary alcohol in the synthetic retinoid CD437 results in a significant reduction of its anticancer activity while maintaining its antimicrobial activity (20).

In addition, based on structure-activity relationship (SAR) studies on synthetic retinoids, we found that the deep penetration of bulky moieties leads to improved antimicrobial activity by enhanced membrane perturbation (20). In the case of nTZDpa, we found that additional halogen substituents and the replacement of chlorine with a larger halogen atom such as iodine significantly enhance antimicrobial activity against both growing and nongrowing antibiotic-tolerant MRSA as well as membrane selectivity for bacterial over mammalian membranes (21). Based on these SAR results, we think the addition of halogen atoms or substitution of larger halogen atoms for chlorine may augment PQ401’s antimicrobial activity. In addition, the SAR studies of diarylurea derivatives aiming at improving biological activity, aqueous solubility, and bioavailability have been intensively conducted and their synthetic procedures are well established (24–26, 77–79). Therefore, the established SAR of diarylurea derivatives coupled with the molecular mechanisms described in this study provides a strong rationale for further optimization of PQ401 as a potential membrane-active antibiotic.

In conclusion, we discovered that PQ401 is a potent antimicrobial that is effective against both multidrug-resistant and multidrug-tolerant S. aureus. PQ401 kills bacteria by selectively disrupting bacterial lipid bilayers, exhibits relatively strong bactericidal activity against growing MRSA cells, has a low probability of selecting for resistance, exhibits synergism with gentamicin against antibiotic-tolerant MRSA, and shows significant efficacy against MRSA in both C. elegans and G. mellonella infection models. Finally, unlike cationic antimicrobial agents, the antimicrobial activity of PQ401 is maximized when it exists in its neutral form. This feature of PQ401 in comparison to cationic antimicrobial peptides significantly expands the potential diversity of membrane-active antimicrobial agents.

## MATERIALS AND METHODS

### Bacterial strains and growth conditions.

Methicillin-susceptible S. aureus strain Newman (80); methicillin-resistant S. aureus (MRSA) strains MW2 BAA-1707 (81) ATCC 33591, and JE2 (82); vancomycin-resistant S. aureus strain VRS1 (32); 11 clinical S. aureus isolates (49); Enterococcus faecium E007 (83, 84); vancomycin-resistant E. faecium strain C68 (34, 85); Klebsiella pneumoniae WGLW2 (BEI Resources, Manassas, VA, USA); Acinetobacter baumannii ATCC 17978 (86); Pseudomonas aeruginosa PA14 (87); and Enterobacter aerogenes ATCC 13048 were used to test antimicrobial activity (Tables 1 to 3). S. aureus strains were grown in tryptic soy broth (TSB) (BD, Franklin Lakes, NJ, USA), and E. faecium strains were grown in brain heart infusion (BHI) broth (BD, Franklin Lakes, NJ, USA) at 37°C at 200 rpm. K. pneumoniae, A. baumannii, P. aeruginosa, and *E. aerogenes* were grown in Luria-Bertani (LB) broth (BD, Franklin Lakes, NJ, USA) at 37°C at 200 rpm.

### Antimicrobial agents and chemicals.

Vancomycin, oxacillin, gentamicin, and ciprofloxacin were purchased from Sigma-Aldrich (St. Louis, MO, USA). PQ401 was purchased from R&D Systems (Minneapolis, MN, USA). Stocks of all antibiotics of 10 mg/ml were made in dimethyl sulfoxide (DMSO) or double-distilled water (ddH_2_O).

### MIC and MBC assays.

The MICs of antibiotics were determined by the standard microdilution method recommended by the Clinical and Laboratory Standards Institute (88). The pH of the medium (cation-adjusted Mueller-Hinton broth) was adjusted to the desired values with NaOH or HCl, and then the media were filter sterilized through 0.22-μm-pore-size membrane filters. The MBC of PQ401 was determined by identifying the lowest concentration that killed ≥99.9% (3 logs) of an initial bacterial inoculum (5 × 10^5^ CFU/ml) in 24 h (89). CFU were determined by serial dilution and spot-plating on tryptic soy agar (TSA) plates. MIC and MBC assays were conducted in triplicate.

### Killing kinetics assay.

An S. aureus overnight culture was diluted 1:10,000 in 25 ml fresh TSB in a 250-ml flask. In order to obtain exponential-phase cells, the diluted cell suspension was incubated at 37°C with shaking at 225 rpm for 4 h until the optical density at 600 nm (OD_600_) reached 0.4 (∼2 × 10^7^ CFU/ml). One milliliter of the exponential-phase cell culture was added to the wells of a 96-well assay block (Corning Costar 3960) containing 1 ml of prewarmed TSB with twice the desired concentrations of compounds. The assay block was sealed with a gas-permeable membrane (Breathe-Easy; Diversified Biotech) and was incubated at 37°C shaking at 200 rpm. At hourly intervals, 400-μl aliquots were taken, washed once with PBS, serially diluted, and spot-plated onto tryptic soy agar (TSA; BD) plates. After incubation at 37°C overnight, the number of cells was calculated based on colony count. These experiments were conducted in triplicate.

### Resistance selection.

Development of resistant mutants by serial passage was conducted as previously described (36). Briefly, an extended range of titers of PQ401 was generated by 2-fold serial dilution with cation-adjusted Mueller-Hinton (CaMH) broth (Difco, Detroit, MI, USA) from three different starting concentrations (20, 24, and 32 μg/ml) covering 0.1875 to 32.0 μg/ml. Three sets of an extended gradient of PQ401 titers were created in a 96-well plate to provide triplicates for the experiment. The same extended range of concentrations of ciprofloxacin was used as a positive control. MRSA MW2 overnight cultures were adjusted to 1 × 10^6^ CFU/ml in CaMH broth, and 50 μl of the diluted cultures was dispensed into the 96-well plates containing 50 μl of the extended gradient of antibiotics. After incubating the plate at 37°C for 24 h, OD_600_ was measured by a spectrophotometer (SpectraMax M2; Molecular Devices). Bacterial growth was defined as OD_600_ of ≥0.1. Bacterial culture at the highest drug concentration that permitted bacterial growth was diluted 1,000-fold in CaMH, and the diluted culture was then used as inoculum for the next passage. The rest of the culture was stored in 16% glycerol at −80°C. This process was repeated for 25 days.

### Antibiotic-tolerant MRSA-killing assay.

As previously demonstrated, 100% of S. aureus cells in a liquid culture become antibiotic-tolerant cells when grown to stationary phase (3–5, 8). Consistently, we have shown previously that when grown to stationary phase, MRSA MW2 is tolerant to conventional antibiotics such as gentamicin, ciprofloxacin, vancomycin, linezolid, and daptomycin (6, 19–21). The antibiotic-tolerant cells of MRSA MW2 were prepared by growing cultures overnight to stationary phase at 37°C at 200 rpm and washing three times with PBS. One milliliter of ∼1 × 10^8^ CFU/ml antibiotic-tolerant MRSA cells was added to 1 ml of PBS containing a 2-fold-higher concentration of the desired concentration of antibiotics in a 96-well assay block (Corning Costar 3960). One milliliter of the antibiotic-tolerant MRSA cell suspension containing appropriate concentrations of antibiotics was added to the wells of a 2-ml deep-well assay block (Corning Costar 3960) and incubated at 37°C, with shaking at 225 rpm. At every hour, 400-μl samples were removed, washed once with PBS, serially diluted, and spot-plated on TSA plates. Colonies were counted after overnight incubation at 37°C to determine the titer of live cells. These experiments were conducted in triplicate.

### SYTOX Green membrane permeability assay.

The pH of phosphate-buffered saline (PBS) was adjusted to the desired values with NaOH or HCl, and then the PBS was filter sterilized through 0.22-μm-pore-size membrane filters. Black, clear-bottom, 96-well plates (Corning no. 3904) were filled with 50 μl of PBS/well containing the indicated concentration of antibiotics. Exponential-phase or stationary-phase antibiotic-tolerant S. aureus MW2 cells prepared as described under “Killing kinetics assay” and “Antibiotic-tolerant MRSA-killing assay,” respectively, were then washed 3 times with the same volume of PBS. The washed cells were adjusted to an OD_600_ of 0.4 (∼2 × 10^7^ CFU/ml) with PBS. SYTOX Green (Molecular Probes) was added to 10 ml of the diluted bacterial suspension to a final concentration of 5 μM and incubated for 30 min at room temperature in the dark. Fifty microliters of the bacterium-SYTOX Green mixture was added to each well of the 96-well plates containing antibiotics. Fluorescence was measured at room temperature using a spectrophotometer (SpectraMax M2; Molecular Devices) at the excitation of 485 nm and the emission of 525 nm. All experiments were conducted in triplicate.

### Preparation of GUVs and observation of effects of compounds on GUVs.

Giant unilamellar vesicles (GUVs) were prepared by the electroformation method described previously (6) with slight modifications. 1,2-Dioleoyl-*sn*-glycero-3-phosphocholine (DOPC), 1,2-dioleoyl-*sn*-glycero-3-phospho-(1′-*rac*-glycerol) (DOPG), 1-palmitoyl-2-oleoyl-glycero-3-phosphocholine (POPC), cholesterol (ovine wool), and 1,2-dioleoyl-*sn*-glycero-3-phosphoethanolamine-*N*-(lissamine rhodamine B sulfonyl) (18:1 Liss Rhod PE) were purchased from Avanti Polar Lipids (Alabaster, AL, USA). Lipid mixtures of 4 mM consisting of DOPC-DOPG-18:1 Liss Rhod PE (7:3:0.005) and POPC-cholesterol-18:1 Liss Rhod PE (7:3:0.005) were dissolved in chloroform, respectively. The procedure for electroformation of both types of GUVs (7DOPC/3DOPG and 7POPC/3cholesterol) was the same. Indium tin oxide (ITO)-coated slides (50 × 75 × 1.1 mm; Delta Technologies, Loveland, CO, USA) were coated with 20 μl of the lipid mixture and dried in a vacuum chamber for 2 h to remove chloroform. An electroformation chamber was made by placing a 2-mm-thick Teflon spacer between the two lipid-coated ITO slides. After adding 2 ml of 100 mM sucrose into the electroformation chamber, the chamber was sealed with binder clips and then connected to an AC field function generator. An AC field of 1.5-V voltage and 10-Hz frequency was applied for 2 h at room temperature, resulting in 10- to 50-μm-sized vesicles. The harvested GUV suspension was diluted (1:3) in a solution containing 1 volume of 100 mM sucrose and 6 volumes of 110 mM glucose. Forty-nine microliters of the diluted GUV suspension (∼100 vesicles) was added into a black, clear-bottom 384-well plate (Corning no. 3712). The plate was left in the dark at room temperature for 15 min until all GUVs settled on the bottom of the plates. After adding 1 μl of compound solution to a well (final compound concentration, 1× MIC), the GUVs were observed and imaged with an optical microscope equipped with fluorescence contrast and a digital camera (63× objectives; Axio Observer A1 and AxioCam MRm; Zeiss, Germany). Images and movies are representative of five independent experiments.

### Human blood hemolysis.

Ten percent human erythrocytes were purchased from Rockland Immunochemicals (Limerick, PA, USA). The erythrocytes were diluted to 4% with PBS, and 100 μl was added to 100 μl of 2-fold serial dilutions of compounds in PBS, 0.2% DMSO (negative control), or 2% Triton X-100 (positive control) in a 96-well plate. The 96-well plate was incubated at 37°C for 1 h and then centrifuged at 500 × *g* for 5 min. One hundred microliters of the supernatant was transferred to a fresh 96-well plate, and absorbance of supernatants was measured at 540 nm. Percent hemolysis was calculated using the following equation: (*A*_540_ of compound-treated sample − *A*_540_ of 0.1% DMSO-treated sample)/(*A*_540_ of 1% Triton X-100-treated sample − *A*_540_ of 0.1% DMSO-treated sample) × 100. All experiments were independently repeated 3 times using different batches of human erythrocytes.

### All-atom MD simulations.

All-atom MD simulations based on the GROMACS package (90) were performed to investigate the interactions between PQ401 or its different ionized forms and a simulated bacterial plasma membrane. The topologies and parameters of the compounds were generated by Automated Topology Builder (54, 55). The bacterial membrane was represented by a mixed lipid bilayer composed of 88 neutrally charged DOPC and 40 negatively charged DOPG lipids (∼7:3 ratio) with Berger’s lipid force field (91), which has been extensively used in previous studies (38, 46–48). Similarly to our previous studies (6, 20, 21), water was represented by the SPC/E model (92); a geometric combining rule was adopted for nonbonded interactions of the compounds with lipids, ions, and water (93, 94); the fast smooth particle-mesh Ewald method (95) was used to calculate the long-rang electrostatic interactions; and sodium ions were added to neutralize the system. The simulation box had an initial size of 5.96 × 5.96 × 12.3 nm, which was large enough to prevent the compounds interacting with their periodic images. The system was modeled as an NPT ensemble (1 atm, 300 K) with periodic boundary conditions in all directions. The time step was 2 fs. After a 500-ns initial equilibration of solvated lipid systems, the compound was introduced into the water phase above the membrane. After 100 ns of reequilibration, the compound was released, and the system was further simulated for 500 ns (96). Additional simulations were performed to get the free energy profiles for the penetrations of compounds into the membrane, which were calculated by steered molecular dynamics (97), umbrella sampling, and the weighted histogram analysis methods (WHAM) (56, 98). In the sampling, the width of each window was 0.15 nm, each window was simulated for 25 ns, and the first 5 ns was discarded in the WHAM analysis.

### C. elegans-MRSA liquid killing assay.

The C. elegans-MRSA infection assay was conducted as described in a previous study (99). A C. elegans *glp-4*(*bn2*)*;sek-1*(*km4*) double mutant strain was used for this assay. The *glp-4(bn2)* mutation blocks the production of progeny at 25°C (100), which prevents matricidal killing during the infection due to internal hatching of eggs (101). The *sek-1(km4)* mutation immunocompromises the worms and increases their sensitivity to infection, which reduces the assay time (102). The worms were maintained at 15°C on 10-cm slow-kill (SK) agar plates seeded with Escherichia coli HB101 (103). Eggs isolated from gravid adults by hypochlorite treatment were resuspended in M9 buffer and incubated with gentle rocking at 15°C for 48 h. Approximately 4,500 L1 hatchlings were placed on each SK plate seeded with HB101 for 52 h at 25°C until the animals grew into sterile young adults. The wells of a black, clear-bottom 384-well plate (Corning no. 3712) were filled with 20 μl M9 buffer including the desired concentrations of PQ401, vancomycin, or 1% DMSO (negative control). After adding 15 young adult C. elegans *glp-4*(*bn2*)*;sek-1*(*km4*) animals to each well of the plate using a COPAS large particle sorter (Union Biometrica, MA, USA), 35 μl of MRSA MW2 suspension was added (OD_600_, 0.08). The assay plate was sealed with a gas-permeable membrane (Breathe-Easy; Diversified Biotech, Dedham, MA, USA) and then incubated in a humidified chamber at 25°C for 5 days. After washing the plate 8 times with M9 buffer using a microplate washer (BioTek ELx405; BioTek, VT, USA), worms were stained with 0.7 μM SYTOX Orange. To evaluate worm survivability, the worms were imaged using an Image Xpress Micro automated microscope (Molecular Devices), capturing both transmitted light and tetramethyl rhodamine isocyanate (TRITC) (535-nm excitation, 610-nm emission) fluorescent images with a 2× objective. Only dead worms are stained by SYTOX Orange. The assay was conducted in biological triplicate.

### G. mellonella survival assays.

Larvae were purchased from a commercial vendor (Vanderhorst, Inc., St. Mary’s, OH, USA) and stored at room temperature in the dark. Upon arrival, 16 randomly healthy larvae between ∼200 and 250 mg were selected for the experiment. An MRSA MW2 overnight culture was washed with PBS twice and then diluted in PBS to ∼1 × 10^8^ CFU/ml. A 10-μl inoculum was injected into the rear left proleg of the larvae using a 10-μl Hamilton syringe (Sigma-Aldrich; catalog no. 24574). Before injection, the syringe was washed with 10% bleach, 70% ethanol, water, and PBS. This washing step was repeated after every six larva injections. After 1 h, 10 μl PBS containing 20-mg/kg (0.5 mg/ml) PQ401 or 25-mg/kg (0.625 mg/ml) vancomycin was administered into the rear right proleg. The wax moths were incubated at 37°C. Survival was monitored every 24 h for up to 120 h. Statistical analyses (Kaplan-Meier survival analysis and with log rank test) were conducted using GraphPad Prism version 8 (GraphPad Software, La Jolla, CA, USA). A *P* value of less than 0.05 was considered significant.
