# Supplementary material for: The Neutrally Charged Diarylurea Compound PQ401 Kills Antibiotic-Resistant and Antibiotic-Tolerant Staphylococcus aureus
Source: mBio. 2020 Jun 30;11(3):e01140-20. doi: 10.1128/mBio.01140-20 (PMC7327171; doi:10.1128/mBio.01140-20)
Supplement: FIG S1 [file mBio.01140-20-sf001.pdf]

Supplementary Figure for

The Neutrally Charged Diarylurea Compound PQ401 Kills Antibiotic Resistant and Antibiotic Tolerant *Staphylococcus aureus*

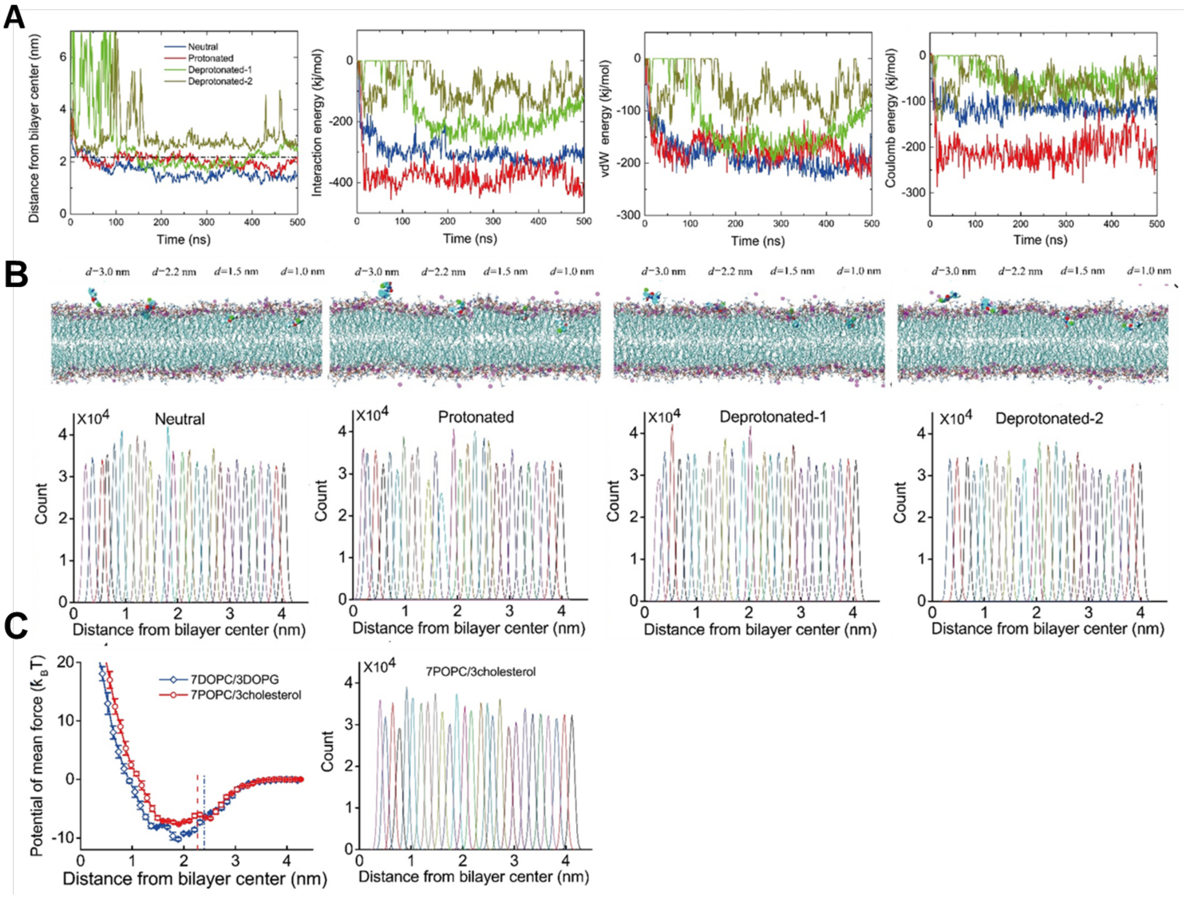

FIG S1 (A) Time evolutions of the distance, the interaction energy and the corresponding Van der Waals and Coulomb interaction energy between PQ401 in different ionized states and the bacterial membrane in MD simulations. (B) Selected snapshots from umbrella simulations with a reference distance about 3.0 nm, 2.2 nm, 1.5 nm and 1.0 nm and the histograms of the umbrella sampling. (C) Free-energy profiles of PQ401 penetrating into the indicated lipid bilayers as a function of the center-of-mass (COM) distance to the bilayer and the corresponding histogram of umbrella sampling. The dot-dashed lines mark the surface of membrane, averaged from the COM locations of phosphate groups in the lipids of the outer leaflet.
